# The speciation and genotyping of *Cronobacter* isolates from hospitalised patients

**DOI:** 10.1007/s10096-015-2440-8

**Published:** 2015-07-15

**Authors:** A. Alsonosi, S. Hariri, M. Kajsík, M. Oriešková, V. Hanulík, M. Röderová, J. Petrželová, H. Kollárová, H. Drahovská, S. Forsythe, O. Holý

**Affiliations:** Pathogen Research Group, School of Science and Technology, Nottingham Trent University, Clifton Lane, Nottingham, NG11 8NS UK; Department of Molecular Biology, Faculty of Natural Sciences, Comenius University, Bratislava, Slovakia; Department of Microbiology, Faculty of Medicine and Dentistry, Palacký University, Olomouc, Olomouc, Czech Republic; Department of Preventive Medicine, Faculty of Medicine and Dentistry, Palacký University, Olomouc, Olomouc, Czech Republic

## Abstract

The World Health Organization (WHO) has recognised all *Cronobacter* species as human pathogens. Among premature neonates and immunocompromised infants, these infections can be life-threatening, with clinical presentations of septicaemia, meningitis and necrotising enterocolitis. The neurological sequelae can be permanent and the mortality rate as high as 40–80 %. Despite the highlighted issues of neonatal infections, the majority of *Cronobacter* infections are in the elderly population suffering from serious underlying disease or malignancy and include wound and urinary tract infections, osteomyelitis, bacteraemia and septicaemia. However, no age profiling studies have speciated or genotyped the *Cronobacter* isolates. A clinical collection of 51 *Cronobacter* strains from two hospitals were speciated and genotyped using 7-loci multilocus sequence typing (MLST), *rpoB* gene sequence analysis, O-antigen typing and pulsed-field gel electrophoresis (PFGE). The isolates were predominated by *C. sakazakii* sequence type 4 (63 %, 32/51) and *C. malonaticus* sequence type 7 (33 %, 17/51). These had been isolated from throat and sputum samples of all age groups, as well as recal and faecal swabs. There was no apparent relatedness between the age of the patient and the *Cronobacter* species isolated. Despite the high clonality of *Cronobacter*, PFGE profiles differentiated strains across the sequence types into 15 pulsotypes. There was almost complete agreement between O-antigen typing and *rpoB* gene sequence analysis and MLST profiling. This study shows the value of applying MLST to bacterial population studies with strains from two patient cohorts, combined with PFGE for further discrimination of strains.

## Introduction

The *Cronobacter* genus belongs to the family Enterobacteriaceae and consists of seven species: *C. sakazakii*, *C. malonaticus*, *C. muytjensii*, *C. turicensis*, *C. dublinensis*, *C. universalis* and *C. condimenti* [1, 2]. In 2002, the International Commission on Microbiological Specifications for Foods (ICMSF) classified *Cronobacter* as pathogenic organisms to a restricted population, endangering their lives and causing serious long-term consequences [3]. The World Health Organization (WHO) has recognised all *Cronobacter* species as microorganisms pathogenic for human beings [4]. Among premature neonates and immunocompromised infants, these infections can be life-threatening, with clinical presentations of septicaemia, meningitis and necrotising enterocolitis. The neurological sequelae can be permanent and the mortality rate can be as high as 40–80 % [5]. Despite the highlighted issues of neonatal infections, the majority of *Cronobacter* infections are in the adult population, especially those suffering from serious underlying disease or malignancy [6]. *Cronobacter* species are also part of the normal flora carriage [7–9].

The first reported age-profiled data was for 819 *Cronobacter* bacteraemia cases in England and Wales between 1992 and 2007 [4]. The majority (91 %) of bacteraemia cases were patients >15 years in age. Holý et al. reported the age profile of *Cronobacter* carriage from a survey of >45,000 patients from two hospitals sampled from 2005 to 2011 [9]. The organism was isolated from every age group, with a higher frequency in children less than 14 years of age. The majority of *Cronobacter* spp. isolates were from throat swabs, followed by urine, tracheal aspirates, bronchoalveolar lavage, cannulae and sputum samples. Patrick et al. also reported an age profile for *Cronobacter* infections from an earlier period (2003–2009), which confirmed its prominence in the adult population, especially in urinary tract infections (UTIs) [6]. However, none of these age profiling studies speciated or genotyped the *Cronobacter* isolates. To date, over 1000 *Cronobacter* strains have been genotyped according to a 7-loci multilocus sequence typing (MLST) scheme [10]. This genotyping has revealed a prevalence of *C. sakazakii* clonal complex 4 with neonatal meningitis cases and *C. malonaticus* clonal complex 7 with adult infections [10–12]. Whole genome phylogenetic analysis (164 genomes) has confirmed the use of *fusA* for *Cronobacter* speciation [10, 13].

This study aimed to address this lack of knowledge using the collection of 51 clinical *Cronobacter* strains, which included those from the study by Holý et al. [9]. These strains were speciated and genotyped using 7-loci MLST, *rpoB* gene sequence analysis, O-antigen typing and pulsed-field gel electrophoresis (PFGE).

## Materials and methods

### Bacterial strains and cultivation

Fifty-one clinical *Cronobacter* strains were used in this study. The strains had been collected during a survey of *Cronobacter* carriage by patients from two hospitals, during a 6-year period from May 2007 to August 2013. This includes strains isolated in the previous study by Holý et al. [9]. Patient information such as age, sex, clinical presentation, isolated site and date of isolation are given in Table 1. Bacterial strains were routinely cultivated on tryptone soya agar (Fluka, UK) at 37 °C overnight.

**Table 1:** Source of *Cronobacter* strains used in this study

Strain number	Hospital	Department	Patient age (years)	Patient sex	Isolation date	Isolation site
1830	Olomouc	Paediatrics	<1	Male	09/05/2007	Throat swab
1829	Olomouc	Paediatrics	1	Male	04/06/2007	Throat swab
1828	Olomouc	Paediatrics	2	Male	12/10/2007	Nose swab
1831	Olomouc	Paediatrics	3	Male	06/06/2007	Throat swab
1832	Olomouc	Paediatrics	3	Female	27/03/2009	Throat swab
1999	Olomouc	Paediatrics	3	Male	30/01/2013	Throat swab
2020	Olomouc	Paediatrics	5	Female	26/05/2013	Stool
1835	Olomouc	Paediatrics	6	Male	30/03/2012	Throat swab
2015	Olomouc	Paediatrics	7	Female	16/08/2013	Throat swab
2014	Olomouc	Paediatrics	8	Male	08/04/2013	Throat swab
1917	Olomouc	Paediatrics	15	Male	28/10/2012	Throat swab
1834	Olomouc	Paediatrics	16	Male	31/05/2010	Throat swab
2004	Olomouc	Paediatrics	17	Female	02/03/2013	Throat swab
1827	Olomouc	Internal Medicine III	76	Female	09/10/2007	Cannula
1833	Olomouc	CMP^a^	5	Male	11/01/2010	Stool
1838	Olomouc	AICU^b^	63	Female	10/04/2012	Sputum
1998	Prostějov	Internal Medicine (A)	49	Female	22/01/2013	Sputum
2008	Prostějov	Internal Medicine (A)	68	Male	12/03/2013	Sputum
2011	Prostějov	Internal Medicine (A)	68	Male	31/03/2013	USC^d^
2006	Prostějov	Internal Medicine (A)	70	Female	28/02/2013	Sputum
2007	Prostějov	Internal Medicine (A)	70	Female	06/03/2013	Sputum
2022	Prostějov	Internal Medicine (A)	70	Female	06/03/2013	Sputum
1842	Prostějov	Internal Medicine (A)	72	Female	27/06/2012	Sputum
2005	Prostějov	Internal Medicine (A)	73	Female	24/02/2013	Sputum
2021	Prostějov	Internal Medicine (A)	76	Female	07/04/2013	Sputum
1841	Prostějov	Internal Medicine (A)	79	Female	18/06/2012	Sputum
2003	Prostějov	Internal Medicine (A)	83	Male	20/02/2013	Sputum
1915	Prostějov	Internal Medicine (A)	84	Female	18/10/2012	Sputum
1996	Prostějov	Internal Medicine (A)	84	Female	14/01/2013	Sputum
2010	Prostějov-	Internal Medicine (A)	84	Female	12/03/2013	Throat swab
2019	Prostějov	Internal Medicine (A)	87	Male	10/05/2013	Sputum
2001	Prostějov	Internal Medicine (B)	68	Male	29/01/2013	SOC^e^
2000	Prostějov	Internal Medicine (B)	71	Male	03/02/2013	Rectal Swab
2002	Prostějov	Internal Medicine (B)	77	Male	19/02/2013	Sputum
1916	Prostějov	Internal Medicine (B)	84	Male	06/11/2012	Sputum
2013	Prostějov	Internal Medicine (B)	91	Female	04/04/2013	Sputum
2012	Prostějov	Internal Medicine (C)	70	Male	04/04/2013	Sputum
2009	Prostějov	Internal Medicine (C)	77	Female	16/03/2013	Tongue swab
1903	Prostějov	Internal Medicine—ICU	59	Male	24/08/2012	Sputum
1902	Prostějov	Internal Medicine—ICU	69	Male	21/08/2012	Sputum
1901	Prostějov	Internal Medicine—ICU	82	Male	15/08/2012	Sputum
1997	Prostějov	ICU^c^	65	Male	21/01/2013	Sputum
1839	Prostějov	ICU	73	Female	12/06/2012	SPEG^f^
1840	Prostějov	ICU	80	Female	19/06/2012	Sputum
1836	Prostějov	Surgery	63	Male	23/05/2012	Wound swab
1837	Prostějov	Surgery	85	Female	25/05/2012	Wound swab
1914	Prostějov	Infectious Diseases	69	Male	02/10/2012	Sputum
2018	Prostějov	Infectious Diseases	72	Male	05/05/2013	Sputum
2016	Prostějov	AICU	27	Male	18/04/2013	Sputum
2017	Prostějov	AICU	27	Male	22/04/2013	Sputum
1995	Prostějov	Outpatient	50	Male	10/01/2013	Sputum

^a^ *CMP* Clinical and Molecular Pathology

^b^ *AICU* Anaesthesiology and Intensive Care Unit

^c^ *ICU* Intensive Care Unit

^d^ *USC* Urine suction catheter

^e^ *SOC* Swab of the oral cavity

^f^ *SPEG* Smear from area of percutaneous endoscopic gastrostomy

### Phenotyping

*Cronobacter* isolates were phenotyped using the ID 32E kit (bioMérieux), according to the manufacturer’s instructions. The resultant phenotypic profiles were compared to the bioMérieux online database at https://apiweb.biomerieux.com.

### PFGE of *Cronobacter* isolates

PFGE analysis of *Cronobacter* isolates was as previously described by Caubilla-Barron et al. [14] using the two restriction enzymes *Xbal* and *Spel* (Promega, UK). The bands were separated using a CHEF-DR II System (Bio-Rad, Belgium) at 14 °C, 6 V for 20 h with initial and final switch of 5 and 50 s, respectively. The DNA band profiles were analysed using BioNumerics software version 7.1 (Applied Maths, Belgium). The banding patterns obtained from the PFGE for both *XbaI* and *SpeI* were combined within the Bionumerics software and analysed by the unweighted pair-group method using arithmetic averages (UPGMA). Isolates with band similarity values of less than 95 % were considered to be non-clonal [15].

### Molecular serotyping of *Cronobacter* O-antigens

*Cronobacter* serotypes were determined using the multiplex polymerase chain reaction (PCR) assay as described by Jarvis et al. and Sun et al. [16, 17]. The allocated serotypes were uploaded to the *Cronobacter* PubMLST database for open access; http://PubMLST.org/cronobacter/.

### DNA extraction

DNA was extracted from the target strains using the GenElute™ kit (Sigma, UK), according to the manufacturer’s instructions. The DNA concentration was confirmed using a NanoDrop® ND-2000 UV–vis spectrometer (Thermo Scientific, UK), and the DNA was stored at −20 °C for 6 months.

### *rpoB* allele sequence analysis

*rpoB* allele profiling was performed as described by Brady et al. [18]. PCR products were visualised on a 1 % agarose gel stained with SYBR Safe. The PCR product (637 bp) was sequenced and aligned with additional sequences from the *Cronobacter* PubMLST database in MEGA (Molecular Evolutionary Genetics Analysis) software version 5.2 [19] using the ClustalW algorithm. *rpoB* alleles were allocated numbered profiles according to the PubMLST database and were uploaded for open access.

### MLST

MLST was performed as previously described by Baldwin et al. [20] and as given on the *Cronobacter* PubMLST open access database (http://www.pubmlst.org/cronobacter/). The seven housekeeping genes amplified were ATP synthase beta chain (*atpD*), elongation factor G (*fusA*), glutaminyl-tRNA synthetase (*glnS*), glutamate synthase large subunit (*gltB*), DNA gyrase subunit B (*gyrB*), translation initiation factor IF-2 (*infB*) and phosphoenolpyruvate synthase (*ppsA*). For multilocus sequence analysis (MLSA), concatenated sequences (3036 bp total length) were aligned in MEGA version 5.2 using the ClustalW algorithm.

## Results

A total of 51 *Cronobacter* strains were characterised by several phenotyping and genotyping methods. Presumptive identification using ID 32E phenotyping identified 49 isolates as *Enterobacter sakazakii*, one strain (1838) as *Pantoea* spp. and the remaining strain (1841) as *E. cloacae*. Since the bioMérieux ID 32E online database does not recognise the *Cronobacter* genus, the strains could not be further identified using this method.

Using the *fusA* sequence analysis and comparison with the *Cronobacter* PubMLST database identified the 51 strains as primarily *C. sakazakii* (33/51), followed by *C. malonaticus* (17/51) and one *C. muytjensii* strain. The strains were then further genotyped using the 7-loci MLST scheme. This supported the species identification-based *fusA* sequence analysis, and further subtyped the isolates (Table 2). The *C. sakazakii* strains were from two sequence types; ST4 (32/51, 63 %) and ST64 (1/51, 2 %). All the *C. malonaticus* strains were ST7 (17/51, 33 %) and the single *C. muytjensii* isolate was ST28 (2 %).

**Table 2:** Number of isolated *Cronobacter* strains from various hospital departments

Hospital	Department	Number of *Cronobacter* strains isolated
Olomouc	Paediatrics	13
	Internal Medicine	1
	AICU^a^	1
	Pathology	1
Prostějov	Internal Medicine	22
	Internal Medicine—ICU^b^	3
	Surgery	2
	ICU	3
	Infectious Diseases	2
	AICU	2
	Outpatient	1
Total		51

^a^ *AICU* Anaesthesiology and Intensive Care Unit

^b^ *ICU* Intensive Care Unit

The identification of strains using *rpoB* sequence analysis [18] and comparison with *rpoB* sequences in the *Cronobacter* PubMLST database agreed with species designation using *fusA* allele sequence analysis (Table 2). There were four different *rpoB* profiles, 1, 18, 35 and 36, which correlated with their 7-loci sequences types. All *C. sakazakii* ST4 and ST64 strains were *rpoB* profiles 1 and 35, respectively. The *C. malonaticus* ST7 strains were *rpoB* profile 18 and *C. muytjensii* ST28 was *rpoB* profile 36. See Table 2 for more information.

Comparison with serotyping profiling showed a strong correlation between some sequence types and serotypes. O-serotype *C. sakazakii* O:2 corresponded with *C. sakazakii* ST4. The association was not exclusive however, as *C. sakazakii* ST64 (strain 1995) was also serotype *C. sakazakii* O:2. In addition, the serotype of all (*n* = 17) *C. malonaticus* ST7 strains corresponded with the two designated serotypes *C. malonaticus* O:2 and *C. sakazakii* O:6 according to the schemes of Jarvis et al. and Sun et al., respectively [16, 17]. Based on *fusA* speciation, *C. malonaticus* O:2 was given as the serotype for these strains (Table 3). No serotype could be determined for the *C. muytjensii* strain as no PCR products were obtained with either PCR serotyping scheme.

**Table 3:** Speciation and genotyping of *Cronobacter* spp. from two hospitals

Strain	Hospital	Species	Pulsotype	*rpoB* allele	*fusA* allele	Serotype	Sequence type
2021	Prostějov	*C. sakazakii*	12	1	1	CS O:2	ST4
2022	Prostějov	*C. sakazakii*	12	1	1	CS O:2	ST4
1901	Prostějov	*C. sakazakii*	12	1	1	CS O:2	ST4
1915	Prostějov	*C. sakazakii*	12	1	1	CS O:2	ST4
1996	Prostějov	*C. sakazakii*	12	1	1	CS O:2	ST4
1837	Prostějov	*C. sakazakii*	12	1	1	CS O:2	ST4
1841	Prostějov	*C. sakazakii*	12	1	1	CS O:2	ST4
1842	Prostějov	*C. sakazakii*	12	1	1	CS O:2	ST4
2003	Prostějov	*C. sakazakii*	12	1	1	CS O:2	ST4
2005	Prostějov	*C. sakazakii*	12	1	1	CS O:2	ST4
2007	Prostějov	*C. sakazakii*	12	1	1	CS O:2	ST4
2010	Prostějov	*C. sakazakii*	12	1	1	CS O:2	ST4
2016	Prostějov	*C. sakazakii*	12	1	1	CS O:2	ST4
2019	Prostějov	*C. sakazakii*	12	1	1	CS O:2	ST4
2017	Prostějov	*C. sakazakii*	12	1	1	CS O:2	ST4
1916	Prostějov	*C. sakazakii*	7	1	1	CS O:2	ST4
1840	Prostějov	*C. sakazakii*	7	1	1	CS O:2	ST4
2000	Prostějov	*C. sakazakii*	7	1	1	CS O:2	ST4
2001	Prostějov	*C. sakazakii*	7	1	1	CS O:2	ST4
2002	Prostějov	*C. sakazakii*	7	1	1	CS O:2	ST4
2009	Prostějov	*C. sakazakii*	7	1	1	CS O:2	ST4
2011	Prostějov	*C. sakazakii*	7	1	1	CS O:2	ST4
1917	Olomouc	*C. malonaticus*	4	18	7	CMal O:2	ST7
1999	Olomouc	*C. malonaticus*	4	18	7	CMal O:2	ST7
2004	Olomouc	*C. malonaticus*	4	18	7	CMal O:2	ST7
2015	Olomouc	*C. malonaticus*	4	18	7	CMal O:2	ST7
2014	Olomouc	*C. malonaticus*	4	18	7	CMal O:2	ST7
2020	Olomouc	*C. malonaticus*	4	18	7	CMal O:2	ST7
1828	Olomouc	*C. malonaticus*	5	18	7	CMal O:2	ST7
1829	Olomouc	*C. malonaticus*	5	18	7	CMal O:2	ST7
1830	Olomouc	*C. malonaticus*	5	18	7	CMal O:2	ST7
1831	Olomouc	*C. malonaticus*	5	18	7	CMal O:2	ST7
1832	Olomouc	*C. malonaticus*	5	18	7	CMal O:2	ST7
1903	Prostějov	*C. sakazakii*	10	1	1	CS O:2	ST4
1998	Prostějov	*C. sakazakii*	10	1	1	CS O:2	ST4
2006	Prostějov	*C. sakazakii*	10	1	1	CS O:2	ST4
2008	Prostějov	*C. sakazakii*	10	1	1	CS O:2	ST4
1833	Olomouc	*C. malonaticus*	3	18	7	CMal O:2	ST7
1834	Olomouc	*C. malonaticus*	3	18	7	CMal O:2	ST7
1835	Olomouc	*C. malonaticus*	3	18	7	CMal O:2	ST7
1902	Prostějov	*C. sakazakii*	11	1	1	CS O:2	ST4
1997	Prostějov	*C. sakazakii*	11	1	1	CS O:2	ST4
1914	Prostějov	*C. malonaticus*	1	18	7	CMal O:2	ST7
2018	Prostějov	*C. malonaticus*	1	18	7	CMal O:2	ST7
1827	Olomouc	*C. malonaticus*	2	18	7	CMal O:2	ST7
2013	Prostějov	*C. sakazakii*	8	1	1	CS O:2	ST4
2012	Prostějov	*C. sakazakii*	9	1	1	CS O:2	ST4
1839	Prostějov	*C. sakazakii*	13	1	1	CS O:2	ST4
1836	Prostějov	*C. sakazakii*	14	1	1	CS O:2	ST4
1995	Prostějov	*C. sakazakii*	15	35	8	CS O:2	ST64
1838	Olomouc	*C. muytjensii*	6	36	24	No PCR product	ST28

**Fig. 1:**
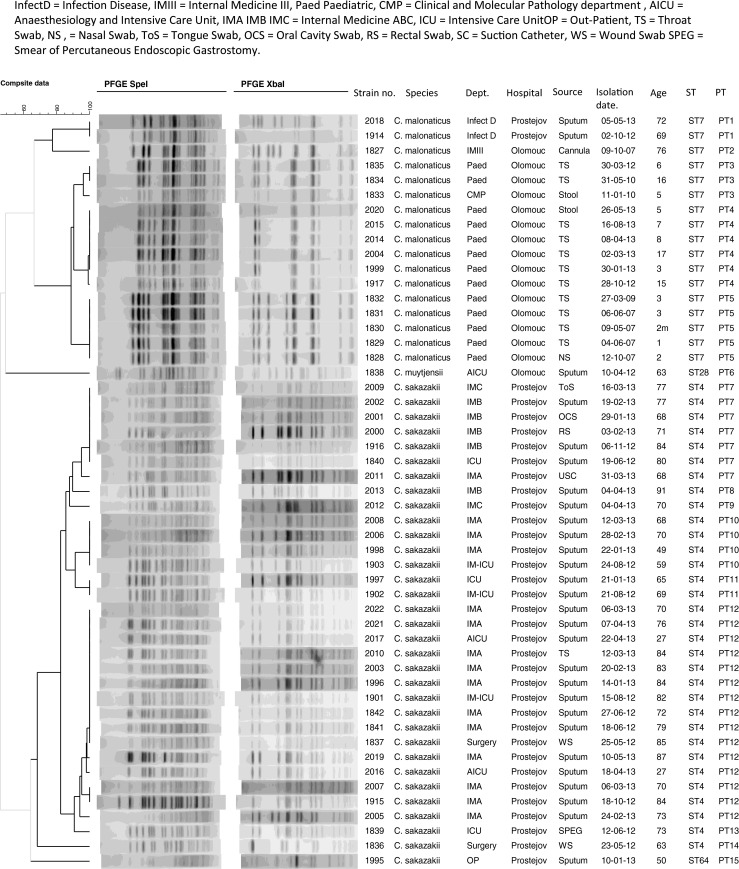
Combined *XbaI* and *SpeI* pulsed-field gel electrophoresis (PFGE) profiles of 51 *Cronobacter* strains. *Infect D* Infectious Diseases, *IMIII* Internal Medicine III, *Paed* Paediatric, *CMP* Clinical and Molecular Pathology, *AICU* Anaesthesiology and Intensive Care Unit, *IMA IMB IMC* Internal Medicine A, B, C, respectively, *ICU* Intensive Care Unit, *OP* Outpatient, *TS* throat swab, *NS* nasal swab, *ToS* tongue swab, *OCS* oral cavity swab, *RS* rectal swab, *USC* urine suction catheter, *WS* wound swab, *SPEG* smear from area of percutaneous endoscopic gastrostomy

PFGE was used to ascertain whether the strains in each sequence type (i.e. *C. sakazakii* ST4 and *C. malonaticus* ST7) could be further distinguished and whether this could be used to profile the strains from the two hospitals. Using the restriction enzyme XbaI, *C. sakazakii* strains gave 12 to 17 DNA fragments per strain, whereas *C. malonaticus* strains gave 8 to 10 bands (Fig. 1). Comparable numbers of fragments were obtained using SpeI: 14 to 17 bands for *C. sakazakii* strains and 14 to 16 bands for *C. malonaticus* strains. The *XbaI* restriction enzyme separated the collection into 16 pulsotypes: ten for *C. sakazakii*, five for *C. malonaticus* and one for *C. muytjensii*, while the *SpeI* restriction enzyme divided the collection into 14 pulsotypes: eight for *C. sakazakii*, five for *C. malonaticus* and one for *C. muytjensii*. Combining the PFGE profiles generated with the restriction enzymes *XbaI* and *SpeI* grouped the 51 strains into a total of 15 pulsotypes: nine for *C. sakazakii*, five for *C. malonaticus* and one for *C. muytjensii*. Strains of the same sequence type from different hospital departments were distinguishable by PFGE and are considered in more detail below.

The isolates from Olomouc hospital formed four distinguishable *C. malonaticus* pulsotypes (PT2 to 5) and one *C. muytjensii* pulsotype (PT6), which were recovered from different age groups of patients from four hospital departments. PT2 was one *C. malonaticus* ST7 strain (1827) isolated in the Internal Medicine Department from the intravenous cannula of a 76-year-old patient in 2007. PT3 was composed of three *C. malonaticus* ST7 strains (1834, 1835, 1833), two of which were isolated from the Paediatric Department and one was from the Clinical and Molecular Pathology Department. The three PT3 strains had been isolated over a 2-year period from throat and stool samples of patients under 16 years of age. The six isolates in PT4 were all *C. malonaticus* ST7 strains. Five had been isolated from the Paediatric Department over a 10-month period from throat swabs and one from a stool sample. The patient ages ranged from 3 to 17 years old. The majority (4/5) of PT5 strains were isolated from the throat and one from nose from the same Paediatric Department. These strains were also *C. malonaticus* ST7 and had been collected over a period of 2 years. The patient ages ranged from 2 months to 3 years. *C. muytjensii* ST28 strain 1838 was in a unique pulsotype (PT6). This strain was isolated in 2012 at the Anaesthesiology and Intensive Care Unit, from the sputum of a 63-year-old patient.

The isolates from Prostějov hospital were recovered from seven departments and were clustered in ten distinguishable *Cronobacter* pulsotypes (Table 3). PT1 was the only *C. malonaticus* pulsotype (strains 1914 and 2018). These were both *C. malonaticus* ST7 strains which were isolated from patients’ sputum at the Infectious Disease Department. The collection was over a 7-month period, and the patients were 69 and 72 years in age. All the remaining isolates were strains of *C. sakazakii*, which formed nine pulsotypes (PT7 to 15). Eight of these pulsotypes (PT7 to 14) were composed of 32 strains of *C. sakazakii* ST4. PT15 was composed of one *C. sakazakii* ST64 strain (1995). Most of the 15 *C. sakazakii* ST4 strains in PT12 were isolated from sputum except strains 1837 and 2010, which were isolated from a wound swab and throat swab, respectively. This pulsotype was collected over period of about 1 year and the patients ages ranged from 27 to 87 years. In PT12, 12 isolates were collected from the Internal Medicine Department, two from the Anaesthesiology and Intensive Care Unit and one from the Surgery Department. PT13 and PT14 each contained single *C. sakazakii* ST4 strains; 1839 and 1836, respectively. PT15 contained a single *C. sakazakii* ST64 strain (1995). These strains were isolated from a percutaneous endoscopic gastrostomy smear ICU, wound surgery and the sputum of an outpatient, respectively. The isolations were over a 7-month period and the patient ages ranged from 50 to 73 years. PT7 consisted of seven *C. sakazakii* ST4; strains 1840, 1916 and 2002 were isolated from sputum, strain 2000 from rectal swab, strain 2001 from oral cavity swab, strain 2009 from tongue swab and strain 2011 from section catheter. Six of the isolates were collected from the Internal Medicine department, and strain 1840 was isolated from an Intensive Care Unit patient. The collection was over a 7-month period and all patients were over 68 years of age. PT8, 9, 10 and 11 consisted of eight *C. sakazakii* ST4 strains. All these strains except one (1997) were isolated from sputum at the Internal Medicine Department, whereas strain 1997 was collected from the Intensive Care Unit. The PT8 strain was isolated in 2013 from a 91-year-old patient. PT9 was isolated in 2013 from a 70-year-old patient. PT10 was collected over a roughly 8-month period and the patient ages were between 49 and 70 years old. The two strains in PT11 were collected in 2012 and 2013 and the mean patient age was 67 years (Table 4).

**Table 4:** Distribution of *Cronobacter* species and genotype according to hospital and patient details

*Cronobacter* species	Sequence type	No. of isolates (%)	Pulsotype (*n*)	Hospital	Period of isolation	Age (years)	Sex		Source (*n*)
							Male	Female	
*C. sakazakii*	ST4	32 (63)	12 (15), 7 (7), 10 (4), 11 (2), 8 (1), 9 (1), 13 (1), 14 (1)	Prostějov	12/06/12–10/05/13	>27	16	16	Sputum (24), wound swab (2), section catheter (1), tongue swab (1), throat swab (1), oral cavity (1), rectal swab (1), SPEG^a^ (1)
*C. sakazakii*	ST64	1 (2)	15 (1)	Prostějov	10/01/2013	50	1	0	Sputum (1)
*C. malonaticus*	ST7	17 (33)	4 (6), 5 (5), 3 (3), 2 (1)	Olomouc	06/05/07–16/08/13	<1 to 76	12	5	Throat swab (11), faecal material (2), cannula (1), nasal swab (1)
			1 (2)	Prostějov	2/10/2012 & 5/05/2013	69 and 72	2	0	Sputum (2)
*C. muytjensii*	ST28	1 (2)	6 (1)	Olomouc	10/04/2012	63	0	1	Sputum (1)
Total		51			6 years		29	22	

^a^ *SPEG* Smear from area of percutaneous endoscopic gastrostomy

**Fig. 2:**
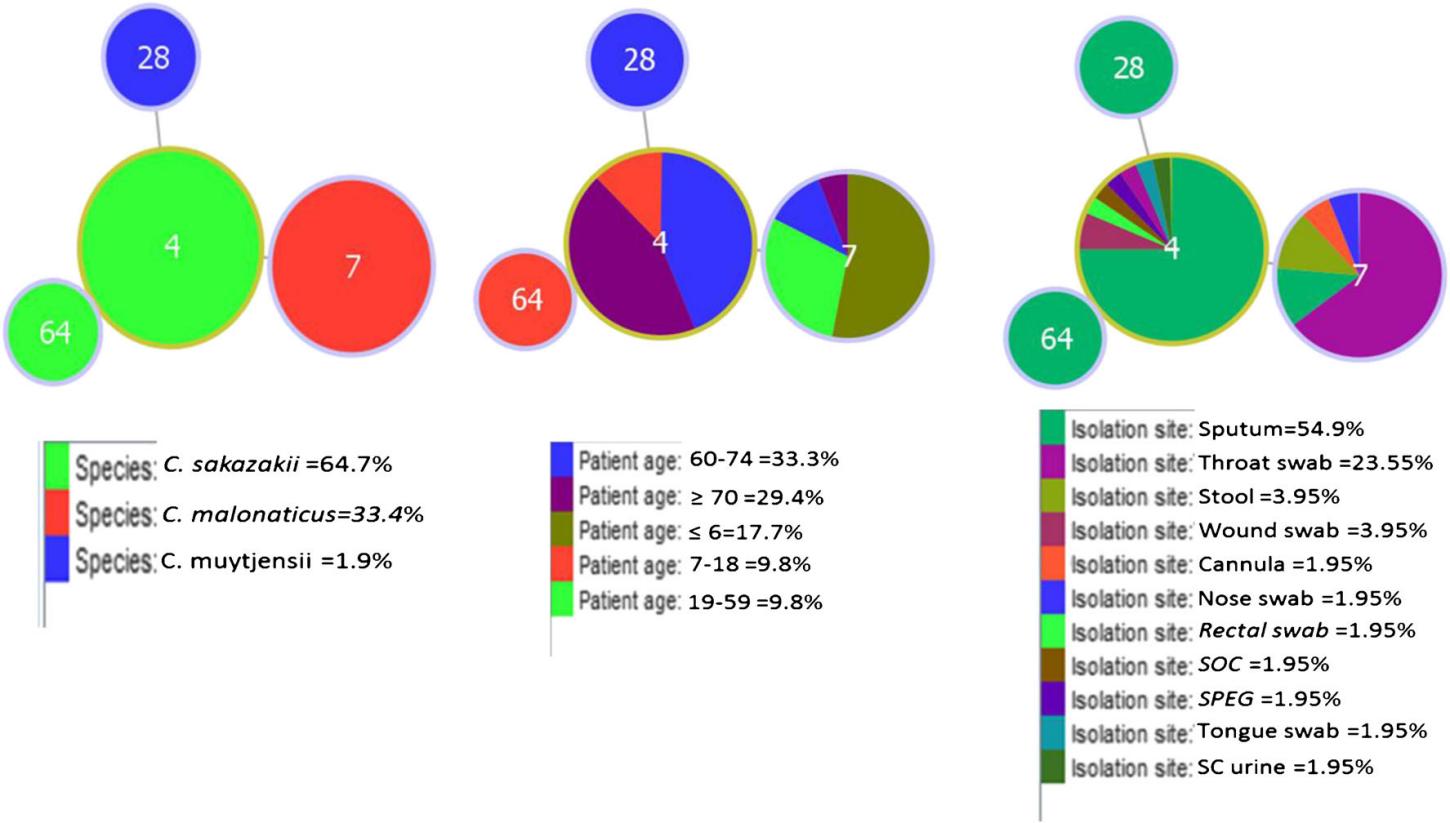
goeBURST analysis of *Cronobacter* strains

goeBURST analysis showed the range of patient ages and sources with *Cronobacter* species (Fig. 2). *C. sakazakii* ST4 strains were predominantly sputum samples from adults >70 years in age, whereas *C. malonaticus* ST7 were from throat swabs of children <6 years old.

## Discussion

Reported *Cronobacter* infections have primarily concerned infants, especially premature neonates with clinical presentations of necrotising enterocolitis and invasive meningitis [21, 22]. Although many of these cases have been linked to contaminated reconstituted infant formula [23], other routes appear to exist, as infections occur in breast-fed infants as well [22, 24, 25]. The carriage of the organism by adults [9] and the high incidence of UTIs [6] indicate that the exposure routes to this bacterium still require further elucidation. In order to have a wider perspective on the exposure to *Cronobacter*, this study speciated and genotyped *Cronobacter* strains from age-profiled clinical isolates, and extended the previous study by Holý et al., who reported the incidence of *Cronobacter* from >45,000 patients [9].

Of the 51 strains, the majority were *C. sakazakii* (65 %) and *C. malonaticus* (33 %) (Table 3). The prominence of these two species in clinical isolates has been previously reported in a review of the international *Cronobacter* PubMLST database with >1000 strains (Forsythe et al. 2014) [10]. *C. sakazakii* ST4 was the predominant sequence type (32/51 strains) and composed all isolates from Prostějov hospital during a 1-year period. Seventeen *C. malonaticus* ST7 strains were isolated from two hospitals, Olomouc and Prostějov, during the 6-year period from 2007 to 2013. Two further strains were identified as ST64 and ST28, which are *C. sakazakii* and *C. muytjensii*, respectively.

PFGE analysis of isolates revealed that the 35 strains isolated at Prostějov hospital could be divided into three groups. The majority (32/35) of strains belonged to *C. sakazakii* ST4 and were serotype *C. sakazakii* O:2. These strains were isolated from various hospital departments during 2012–2013. Two other group isolates were also recovered from patients in this hospital. These were two strains of *C. malonaticus* ST7 and were serotype *C. malonaticus* O:2, and were the only strains isolated from the Department of Infectious Diseases. The remaining strain was *C. sakazakii* ST64 serotype O:2, which was isolated from an outpatient (50 years old, sputum).

In contrast, all but one of the 16 *Cronobacter* strains isolated from patients at Olomouc hospital were *C. malonaticus* ST7 ; the other isolate was *C. muytjensii*. The *C. malonaticus* strains belonged to the identical sequence type 7 and identical serotype *C. malonaticus* O:2. With two exceptions, all these strains were from patients at the Department of Paediatrics and had an age range of 0–18 years. There were two strains from adults, one *C. malonaticus* from an intravenous cannula and another which was *C. muytjensii* from sputum.

Despite the greater discrimination of strains using PFGE than MLST, isolates from patients for whom there were no known links could not be further differentiated. For example, the *C. sakazakii* ST4, pulsotype 12 strains were isolated from 15 adults (aged 27–85 years) between May 2012 and May 2013. This could be due to the reported high clonality of sequence types within *C. sakazakii* and *C. malonaticus* limiting the discriminatory power of PFGE [1, 10].

In summary, these clinical isolates were predominated by *C. sakazakii* ST4 (63 %, 32/51) and *C. malonaticus* ST7 (33 %, 17/51). These had been isolated from throat and sputum samples of all age groups, as well as recal and faecal swabs. There was no apparent relatedness between the age or sex of the patient and the *Cronobacter* species isolated. Despite the high clonality of *Cronobacter*, PFGE profiles differentiated strains within each sequence type into 15 pulsotypes. There was almost complete agreement between O-antigen typing and *rpoB* gene sequence analysis and MLST profiling. The majority (43/51) of strains were from the upper respiratory system (i.e. throat swabs and sputum samples) and only three were from faeces and one from urine; two being *C. sakazakii* ST4 and the remaining two *C. malonaticus* ST7. Hence, it is plausible that this small sampling of the lower intestinal tract and UTIs does not reflect the diversity of *Cronobacter* in those samples. Given the high incidence of *Cronobacter* in UTI, this area needs further consideration [6].

This study shows the value of applying MLST to bacterial population studies with strains from two patient cohorts, combined with PFGE for further discrimination of strains.
